# Glucagon-like peptide-1 receptor overexpression in cancer and its impact on clinical applications

**DOI:** 10.3389/fendo.2012.00158

**Published:** 2012-12-06

**Authors:** Meike Körner, Emanuel Christ, Damian Wild, Jean Claude Reubi

**Affiliations:** ^1^Division of Cell Biology and Experimental Research, Institute of Pathology, University of BerneBerne, Switzerland; ^2^Division of Endocrinology, Diabetology and Clinical Nutrition, University Hospital of BerneInselspital, Berne, Switzerland; ^3^Division of Nuclear Medicine, Department of Radiology, University Basel HospitalBasel, Switzerland

**Keywords:** glucagon-like peptide-1, glucagon-like peptide-1 receptor, insulinoma, ^111^In-DOTA/DPTA-exendin-4

## Abstract

Peptide hormones of the glucagon-like peptide (GLP) family play an increasing clinical role, such as GLP-1 in diabetes therapy. Moreover, GLP receptors are overexpressed in various human tumor types and therefore represent molecular targets for important clinical applications. In particular, virtually all benign insulinomas highly overexpress GLP-1 receptors (GLP-1R). Targeting GLP-1R with the stable GLP-1 analogs ^111^In-DOTA/DPTA-exendin-4 offers a new approach to successfully localize these small tumors. This non-invasive technique has the potential to replace the invasive localization of insulinomas by selective arterial stimulation and venous sampling. Malignant insulinomas, in contrast to their benign counterparts, express GLP-1R in only one-third of the cases, while they more often express the somatostatin type 2 receptors. Importantly, one of the two receptors appears to be always expressed in malignant insulinomas. The GLP-1R overexpression in selected cancers is worth to be kept in mind with regard to the increasing use of GLP-1 analogs for diabetes therapy. While the functional role of GLP-1R in neoplasia is not known yet, it may be safe to monitor patients undergoing GLP-1 therapy carefully.

## INTRODUCTION

G protein-coupled peptide hormone receptors play an increasing role as tumor targets in cancer medicine (Reubi, 2003). The underlying molecular basis is primarily an overexpression of a specific peptide receptor on tumor cells, irrespective of receptor functions. This overexpression allows a receptor-targeted scintigraphic imaging and radiotherapy of tumors with adequate radiolabeled peptide analogs (Reubi, 2003). Historically, the somatostatin receptors were the first receptors identified for these purposes (Reubi, 2003). They are expressed in high incidence and at high levels in gastroenteropancreatic neuroendocrine tumors (Reubi, 2003). Somatostatin receptor scintigraphy using the somatostatin analog octreotide (OctreoScan^®^) represents nowadays a standard imaging procedure for patients with gut neuroendocrine tumors, while PET/CT with ^68^Ga-labeled somatostatin analogs turns out to be even superior to OctreoScan^®^ (Gabriel et al., 2007). Furthermore, results from clinical studies performing somatostatin receptor-mediated radionuclide therapy are encouraging (Kwekkeboom et al., 2008; Imhof et al., 2011).

The clinical success of somatostatin receptor targeting of gut neuroendocrine tumors has stimulated the search for other peptide receptors suitable for similar applications. A promising candidate is the glucagon-like peptide 1 (GLP-1) receptor (GLP-1R). This receptor has been cloned almost 20 years ago (Thorens et al., 1993). It is a member of the class 2 G protein-coupled receptor family (Gros et al., 1993; Nauck, 2009). Only a single GLP-1R has been identified so far, which is structurally identical in all tissues (Thorens et al., 1993). Receptor activation upon agonist binding stimulates adenylate cyclase and phospholipase C, with subsequent activation of protein kinase A and C, respectively (Thorens et al., 1993).

Physiologically, the GLP-1R is expressed mainly in the alimentary tract, particularly in the pancreatic islet cells (Wei and Mojsov, 1995) where it mediates the actions of GLP-1 released from the small intestines in response to food intake. GLP-1 is considered to be one of the most important glucose-dependent insulin secretagogues (Nauck, 2009). Specifically, it stimulates glucose-dependent insulin synthesis and secretion, inhibits glucagon secretion, decreases β-cell apoptosis and increases differentiation of β-cell precursor cells in the pancreas as well as inhibits gastric emptying and appetite at the hypothalamic level (Nauck et al., 2009). Exploiting GLP-1 pathways therefore represents an ideal therapeutic approach for patients with type 2 diabetes, as this interferes with the main pathophysiological mechanisms of the disease (Nauck et al., 2009). Indeed, synthetic GLP-1 analogs are FDA- and EMEA-approved for the treatment of type 2 diabetes (Nauck et al., 2009).

The GLP-1R is of clinical interest not only due to its physiologic expression and functions in pancreatic islet cells and its potential in diabetes therapy, but also because of its possible role in cancer. Indeed, 10 years ago, the GLP-1R was found to be expressed in insulin-producing islet cell tumors, i.e., insulinomas (Reubi and Waser, 2003). This discovery lead to an extensive evaluation of the potential of the GLP-1R for targeted tumor imaging and therapy analogous to somatostatin receptor targeting of gut neuroendocrine tumors. This evaluation included the characterization of human tumors and normal tissues for their GLP-1R expression, since an important prerequisite for a successful peptide receptor targeting of tumors is a high receptor expression in tumors, but a low receptor expression in normal background tissues. Further activities included the development of adequate radiolabeled GLP-1 analogs, testing of such analogs in *in vivo* animal models and application of selected suitable candidate analogs to tumor patients in preliminary clinical studies. This review summarizes the knowledge on the *in vitro* and *in vivo* basis of GLP-1 receptor targeting of tumors accumulated in the last decade.

## GLP-1R IN TUMORS

**FIGURE 1:**
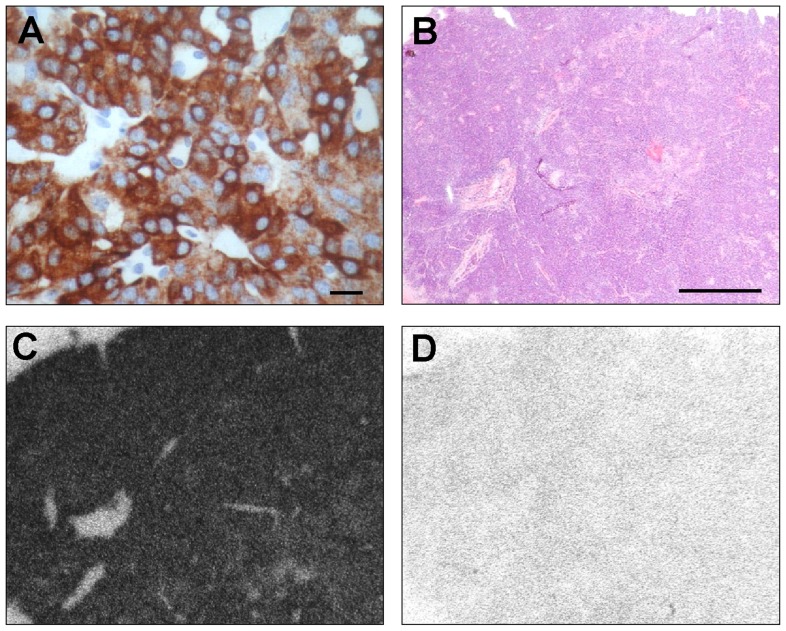
**Hormone and receptor determinations *in vitro* in a benign insulinoma**. **(A)** Immunohistochemistry for insulin showing strongly labeled tumor cells. Bar = 0.01 mm. **(B–D)** *In vitro* GLP-1R autoradiography on consecutive insulinoma tissue sections. **(B)** Hematoxylin & eosin (H&E) staining showing the tumor tissue. Bar = 1 mm. **(C)** Autoradiogram showing total binding of ^125^I-GLP-1(7-36) amide. The entire tumor is strongly positive. **(D)** Autoradiogram showing non-specific binding of ^125^I-GLP-1(7-36) amide in the presence of 100 nM cold GLP1(7-36) amide. Reprinted from Christ et al. (2010), with permission from Elsevier.

The GLP-1R expression has been systematically assessed in a broad spectrum of original human tumor tissues using *in vitro* receptor autoradiography (Reubi and Waser, 2003; Korner et al., 2007; Waser et al., 2011). The GLP-1R was thus identified in specific endocrine, embryonal, and brain tumors, but virtually not in carcinomas (**Table 1**). The most striking GLP-1R expression was found in insulinoma. This is an endocrine tumor of the pancreatic islet cells with mostly benign biological behavior, but characterized clinically by severe symptoms of hyperinsulinism due to insulin secretion. Benign insulinomas expressed GLP-1Rs in very high incidence (>90%) and extremely high density (Reubi and Waser, 2003; **Table 1**; **Figure 1**). In fact, no other peptide receptor has been found to exhibit such high expression levels in this tumor type (Reubi and Waser, 2003). On the contrary, malignant, metastasizing insulinomas expressed GLP-1Rs significantly less frequently. High GLP-1R levels were found in only 36% malignant insulinomas (Wild et al., 2011). In insulinoma cells, the GLP-1R may represent a mediator of insulin secretion: in a model of GLP-1R transfected insulinoma cells, glucose-mediated insulin release was increased compared to control cells, in parallel with an increase of the intracellular second messenger of the GLP-1R (cAMP; Montrose-Rafizadeh et al., 1997).

**Table 1:** GLP-1R expressing human tumors: receptor incidences and densities.

Tumor type	GLP-1R incidence	GLP-1R densityfacilities*
**Endocrine tumors**	
Benign insulinomas	25/27 (93%)	8,133
Malignant insulinomas	4/11 (36%)	8,508facilities^†^
Gastrinomas	10/10 (100%)	2,461
Glucagonomas	2/4 (50%)	910
VIPomas	1/4 (25%)	3,028
Ileal carcinoids	8/27 (30%)	1,027
Bronchial carcinoid tumors	11/29 (38%)	2,456
Pheochromocytomas	12/20 (60%)	3,970
Paragangliomas	5/18 (28%)	1,353
Medullary thyroid carcinomas	5/18 (28%)	1,326
**Embryonal tumors**
Medulloblastomas	3/12 (25%)	1,246
Nephroblastomas	2/9 (22%)	421
Neuroblastomas	3/16 (19%)	932
**Brain tumors**	
Meningiomas	7/20 (35%)	989
Astrocytomas	4/16 (25%)	1,069
Glioblastomas	2/21 (9%)	790
Ependymomas	1/6 (16%)	1,075
**Carcinomas**
Ovarian adenocarcinomas	2/12 (16%)	688
Prostate adenocarcinomas	1/20 (5%)	1,283

* dpm/mg tissue.

† Mean value of two tumors tested with *in vitro* GLP-1R autoradiography

Also several other functioning endocrine tumors of the pancreas expressed GLP-1Rs, in particular gastrinomas, however in lower amounts compared with insulinomas (**Table 1**; Reubi and Waser, 2003). Moreover, GLP-1Rs were discovered in a number of extrapancreatic endocrine tumors, including ileal carcinoids, pheochromocytomas, paragangliomas, bronchial carcinoid tumors, and medullary thyroid carcinomas, while they were not identified in pituitary adenomas or adrenal cortical tumors (Korner et al., 2007). Pheochromocytomas are of particular clinical interest due to their high GLP-1R expression levels (**Table 1**). Furthermore, medullary thyroid carcinomas are noteworthy because of important species differences in their GLP-1R expression. In rats, virtually all medullary thyroid carcinomas expressed GLP-1Rs in high amounts (Waser et al., 2011), while in humans only 28% expressed GLP-1R at low density levels (**Table 1**).

Lower GLP-1R expression levels were found in embryonal tumors, including medulloblastoma, nephroblastoma, and neuroblastoma (**Table 1**). They showed GLP-1Rs in low density in 15–25% of the tumors (Korner et al., 2007). Similarly, tumors of the nervous system such as meningiomas and astrocytomas demonstrated an incidence of GLP-1Rs between 25 and 35%, whereas glioblastomas and ependymomas expressed GLP-1Rs in 9–16% (**Table 1**; Korner et al., 2007). Schwannomas were devoid of GLP-1Rs (Korner et al., 2007).

Conversely, carcinomas exhibited a very low or no GLP-1R expression. Only ovarian and prostate carcinomas rarely showed GLP-1R at low levels, while breast, colorectal, gastric, pancreatic, hepatocellular, and cholangiocellular as well as lung carcinomas (non-small and small cell carcinomas) were negative for GLP-1R (Korner et al., 2007). Likewise, non-Hodgkin lymphomas did not express GLP-1R (Korner et al., 2007).

Among all GLP-1R expressing tumor types, insulinomas are at present of highest clinical interest for an *in vivo* targeting in patients, based on several considerations. First, insulinomas exhibit particularly high GLP-1R expression levels with respect to both incidence and density. Second, benign insulinomas, in contrast to most other gastroenteropancreatic neuroendocrine tumors, show relatively low expression levels of somatostatin receptors. Consequently, OctreoScan^®^ is not a reliable tool to detect these tumors (Plockinger et al., 2004). Third, the exact intraoperative localization of insulinomas is critical in order to minimize the surgical intervention (Rostambeigi and Thompson, 2009). This is, however, difficult due to the small size of benign insulinomas (usually 10–20 mm). Conventional radiological procedures (endosonography, MR-, and CT-imaging) are not always successful in localizing insulinomas (Chatziioannou et al., 2001). Moreover, [^18^F]DOPA PET shows at present controversial results, with sensitivities ranging between 17 and 90% (Kauhanen et al., 2007; Tessonnier et al., 2010). Although selective arterial stimulation and venous sampling is a reliable intraoperative tool to detect insulinomas in experienced institutions (Wiesli et al., 2004), it is an invasive procedure with the associated risks. Moreover, this procedure identifies only the region of the pancreas – depending on the vasculature – where the insulinoma should be located and not the tumor itself (Wiesli et al., 2004).

## GLP-1R IN NON-NEOPLASTIC TISSUES

The GLP-1R expression was similarly characterized in human normal tissues. It has been found in the pancreatic islets and acini, stomach, duodenal Brunner’s gland, small and large intestinal myenteric plexus, lung and kidney vasculature, breast parenchyma, heart, brainstem, hypothalamus, neurohypophysis, and meninges (Wei and Mojsov, 1995; Korner et al., 2007). *In vitro* receptor autoradiography revealed that GLP-1R levels were highest in the neurohypophysis, followed by Brunner’s glands, meninges, and pancreatic islets (Korner et al., 2007). Of practical importance, with the exception of Brunner’s glands, the different tissues in the pancreatic area (i.e., pancreas islets and acini, intestines, and kidney) exhibit far lower GLP-1R density levels than insulinomas. This results in a high tumor-to-background ratio in GLP-1R density levels for insulinomas, which is an important prerequisite for a GLP-1R-targeted scintigraphic imaging of insulinomas.

Prominent species differences in the physiological GLP-1R expression between humans and rodents are noteworthy. Indeed, autoradiography experiments indicate that GLP-1R density levels are considerably higher in the lungs of rats and mice than of humans (Korner et al., 2007). This has to be considered when interpreting results of *in vivo* testing of GLP-1R targeting in rodent models. Likewise, the GLP-1R expression in the thyroid gland is substantial in rodents, but virtually absent in humans (Korner et al., 2007). In rodent thyroids, GLP-1Rs are located in the medullary C-cells. Of interest, treatment with GLP-1 analogs in rats is known to occasionally lead to thyroid C-cell hyperplasia and medullary thyroid carcinoma, whereas in humans there is so far no evidence of such complications. It can be speculated whether the species differences in the GLP-1R density expression in the precursor cells of these tumors, the medullary C-cells, contribute to these controversial findings (Waser et al., 2011).

## RADIOLABELED GLP-1 ANALOGS

In general, radioactively labeled peptide analogs represent pharmaceuticals with favorable characteristics. Due to their small size, they show fast diffusion and rapid blood clearance and lack immunogenicity. Moreover, radiopeptides exhibit only rare side effects (Reubi, 2003). In addition, radiolabeling is easily feasible, preferably after attaching a chelator to the peptide (Reubi and Maecke, 2008). However, since peptides are physiologically degraded within minutes in the human blood by potent peptidases such as dipeptidyl-peptidase-4 (DPP-4; Baggio and Drucker, 2007), stable peptide analogs have to be used instead in clinical applications. As for GLP-1, a naturally occurring stable analog exists, namely exendin-4, which is a component of the Gila monster venom. It shares 53% homology with GLP-1 and similarly binds to GLP-1Rs, but is resistant to DPP-4 cleavage (Nauck, 2009). Exendin-4 is, therefore, a good candidate for the development of radiolabeled GLP-1R ligands.

The first radiopeptides tested for *in vivo* GLP-1R targeting were ^125^I-labeled GLP-1 and the GLP-1 analog exendin-3 (Gotthardt et al., 2002). However, the low peptide stability of GLP-1 and the low efficiency of radio-iodination of exendin-3 limited their clinical use. Further testing resulted in the development of ^111^In-labeled exendin-4 (Wild et al., 2006): exendin-4 was coupled via the Lys side chain to a chelator (DOTA, tetraazacyclododecane tetraacetic acid or DTPA, diethylenetriaminepentaacetic acid) using a spacer (Ahx, aminohexanoic acid) and then labeled with ^111^In. This radiopeptide was subsequently extensively tested *in vitro* and *in vivo* in insulinoma models and applied to insulinoma patients (see below). Lately, several studies have been published that describe GLP-1R ligands suitable for PET/CT imaging, such as ^68^Ga-, ^64^Cu-, or ^18^F-labeled exendin-4, or for SPECT/CT imaging like ^99m^Tc-labeled exendin-4 (Brom et al., 2010; Wild et al., 2010; Wu et al., 2011; Kiesewetter et al., 2012). These novel radiopeptides have not yet been tested in insulinoma patients.

## GLP-1R TARGETING IN ANIMAL MODELS

Initially, GLP-1R targeting was performed in the rat insulinoma cell line RINm5F and in a rat insulinoma animal model (NEDH rats) using ^125^I-labeled GLP-1 and exendin-3 (Gotthardt et al., 2002). Specific uptake was detected in the cell and animal models. This provided the proof of principle for GLP-1R targeting of insulinoma (Gotthardt et al., 2002).

Follow-up experiments were carried out in the Rip1tag2 mouse model with ^111^In-DTPA-exendin-4. These transgenic mice develop tumors of the pancreatic β-cells in a reproducible multistage fashion (Hanahan, 1985) and, therefore, represent an ideal model to study GLP-1R targeting *in vivo* and *in vitro*. Using GLP-1R multipinhole SPECT/MRI and SPECT/CT, *in vivo* GLP-1R imaging was performed in these animals following administration of ^111^In-DTPA-exendin-4 (Wild et al., 2006). In parallel, GLP-1R autoradiography of the tumors was carried out *in vitro*. Finally, biodistribution and pharmacokinetics as well as internalization and cellular retention of ^111^In-DTPA-exendin-4 were measured *in vitro* (Wild et al., 2006).

This preclinical study showed the following main findings: First, the GLP-1R density in the tumors was extremely high, resulting in a remarkably high uptake of ^111^In-DTPA-exendin-4 (287 ± 62% IA/g tissue) already 4 h after injection. Second, excellent visualization of tumors as small as 1 mm by pinhole SPECT/MRI and SPECT/CT was demonstrated. Third, the tumor-to-background ratio was very high (between 13.6 and 299), substantiating the high potential of this radiopeptide to specifically localize GLP-1R positive lesions within the pancreas. Lastly, *in vitro* studies in the cells derived from the tumor model demonstrated a specific internalization of ^111^In-DTPA-exendin-4, and biochemical investigations confirmed the high metabolic stability of the radiopeptide in the tumor cells as well as in the serum.

The same Rip1tag2 mouse model also provided preliminary data on GLP-1R-targeted therapy of insulinoma. Mice were injected with different doses of ^111^In-DTPA-exendin-4 (1.1, 5.6, and 28 MBq) and sacrificed 7 days after injection. Most impressively, a single injection lead to a reduction in tumor volume of up to 94% in a dose-dependent manner without significant acute organ toxicity. Histological examination revealed that the decrease in tumor mass was mainly due to an increase in tumor cell apoptosis and decreased proliferation (Wicki et al., 2007).

## GLP-1R TARGETING IN HUMANS

The first patient who underwent GLP-1R scintigraphy suffered from severe endogenous hyperinsulinemic hypoglycemia with non-convulsive seizures. MRI, CT scan, and endosonography did not detect any suspicious lesion. However, GLP-1R scintigraphy revealed an increased extrapancreatic uptake in the mesentery supplied by the anterior mesentery artery. Selective arterial stimulation and venous sampling correctly indicated the vascular territory, but since this patient had an ectopic insulinoma, the results of the invasive investigation without GLP-1R imaging would have been misleading for the surgical strategy (Wild et al., 2008).

In a proof of principle study, ^111^In-DOTA-exendin-4 was prospectively administered to a total of six patients (Christ et al., 2009). All of them presented with neuroglycopenic symptoms lasting for 4–26 months. Biochemical evaluation during a fasting test revealed endogenous hyperinsulinemic hypoglycemia in all patients.

**FIGURE 2:**
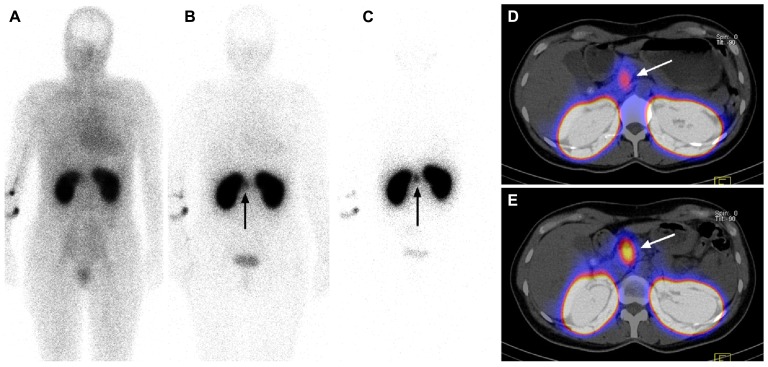
^111^In-DOTA-exendin-4 whole-body planar images **(A–C)** and ^111^In-DOTA-exendin-4 SPECT/CT images **(D,E)** from the same patient. Whole-body scans were carried out 20 min **(A)**, 4 h **(B)**, and 3 days **(C)**, and SPECT/CT scans were performed 4 h **(D)** and 3 days **(E)** after injection of 97 MBq ^111^In-DOTA-exendin-4. Four hours after injection, there was already a focal ^111^In-DOTA-exendin-4 uptake visible in the head of the pancreas (arrow) on whole-body **(B)** and SPECT/CT **(D)** scans. The tumor-to-pancreas-uptake ratio was 1.9 at 4 h after injection **(D)** and 3.2 at 3 days after injection of the radioligand. The longest residence times of ^111^In-DOTA-exendin-4 were observed in the tumor (arrow) and kidneys **(C)**. Reprinted from Christ et al. (2009), with permission from The Endocrine Society.

Conventional imaging (CT or MRI) reliably detected the insulinoma in only two patients, whereas endosonography identified a possible lesion in four patients, in keeping with data in the literature (McAuley et al., 2005). In three patients, selective arterial stimulation and venous sampling was performed, with accurate localization in all (Christ et al., 2009). Remarkably, GLP1-R scintigraphy correctly detected the insulinoma in all six consecutive patients (**Figure 2**; Christ et al., 2009). Four patients underwent an enucleation of the insulinoma. In two patients, a Whipple procedure had to be performed due to the localization of the insulinoma. In all patients, a benign insulinoma was confirmed by histology. *In vitro* autoradiography studies showed GLP-1R densities in the range as previously described (between 2,600 to >10,000 dpm/mg tissue; Reubi and Waser, 2003), but low levels of somatostatin receptor type 1 in 2 patients only (Christ et al., 2009). Importantly, within a time frame of 2–14 days after injection of ^111^In-DOTA-exendin-4, intraoperative utilization of a gamma probe was highly beneficial for the *in situ* localization of the insulinoma in all patients, resulting in a successful enucleation where possible (Christ et al., 2009).

Fortunately, background uptake over the whole body was low with the exceptions of the kidneys, which were strongly labeled due to renal excretion of the radioligand (**Figure 2**). In two patients demarcation between tumors (maximal diameter of 9–11 mm) and kidneys was only possible after late scans, indicating an improved tumor-to-kidney ratio with time, in keeping with the fact that the effective half-life of ^111^In-DOTA-exendin-4 was longer in the tumor (38–64 h) than in the kidneys (31.2–31.8 h; **Figure 2**; Christ et al., 2009). This suggests that patients with negative early scans should have additional imaging 3–7 days after the injection.

In humans, ^111^In-DOTA-exendin-4 was initially administered, but later replaced by ^111^In-DTPA-exendin-4 due to the four times higher specific activity of the latter (Wild et al., 2009). A higher specific activity permits to reduce the amount of peptide (exendin-4), thereby decreasing the occurrence of possible side effects. In addition, radiolabeling of DTPA-exendin-4 can be performed at room temperature, whereas labeling of DOTA-exendin-4 has to be accomplished at high temperature (95°C; Wild et al., 2006).

By now, data of a prospective, multicenter trial including 30 patients that underwent GLP-1R scanning are available. The inclusion criteria were proven endogenous hyperinsulinemic hypoglycemia and none or maximally one lesion on conventional imaging. Conventional imaging (CT, MRI) and endosonography – where available – was performed locally using a standard protocol. ^111^In-DTPA-exendin-4 was administered intravenously at a dose of 90–130 MBq over 2 min. Whole-body planar images and SPECT/CT of the abdomen were performed at 4, 24 and in some patients between 72 and 96 up to 168 h post-injection, the most important time point being the scan 24 h after injection. Dia-gnosis was confirmed by histology after surgical removal (Christ et al., 2012).

Conventional imaging (MRI, CT, endosonography) was positive in 17 patients. ^111^In-DTPA/DOTA-exendin-4 SPECT/CT detected 23 true positive benign insulinomas and five additional positive lesions (one malignant insulinoma; two islet cell hyperplasias; two uncharacterized lesions). True negative tests were detected in two patients (one malignant insulinoma; one islet cell hyperplasia). Malignant insulinomas were diagnosed based on the histological finding of a positive lymph node, not detected on conventional imaging preoperatively. There was no false negative result. Sensitivity was 100% and the positive predictive value was 82% (Christ et al., 2012). These findings are encouraging and suggest that *in vivo* GLP-1R imaging defines a new non-invasive diagnostic approach to successfully localize small benign insulinomas.

About 90% of insulinomas are benign and only 10% of patients present with malignant disease usually characterized by liver metastasis (Plockinger et al., 2004). Anecdotal evidence suggests that malignant insulinomas exhibit more often somatostatin receptors type 2 than benign ones and can, therefore, be visualized by OctreoScan^®^ (Plockinger et al., 2004). A more extensive study with data from 10 patients with malignant insulinoma showed that somatostatin receptors type 2 were expressed in seven patients, whereas GLP-1R were present in four patients, and both receptors in only one patient (Wild et al., 2011). Importantly, one of the two imaging methods appears always to be positive in a malignant type of insulinoma (Wild et al., 2011). The consequences of the respective receptor expression in an insulinoma with regard to biological behavior (malignant or benign course) remains to be established.

## SIDE EFFECTS AND LIMITATIONS

In humans, the injection of ^111^In-DOTA-exendin-4 and ^111^In-DPTA-exendin-4 was well tolerated. Due to the small amount of exendin-4, the decrease in plasma glucose concentrations was only 1.4 ± 0.7 mmol/L after 40 min (Christ et al., 2009). By regularly monitoring glucose levels, no severe hypoglycemic episode occurred. One patient experienced a short episode of vomiting only with ^111^In-DOTA-exendin-4. Otherwise, no further side effects were observed (Christ et al., 2009).

In two patients, there was focal ^111^In-DOTA-exendin-4 uptake in the proximal duodenum. This may be related to the presence of Brunnner’s gland of the duodenum which, as previously mentioned, are known to contain GLP-1Rs in a significant density (Korner et al., 2007). Brunner’s glands may become hyperplastic (Levine et al., 1995), as observed in particular in patients with chronic pancreatitis (Stolte et al., 1981). Such hyperplastic glands may possibly be detected by GLP-1R imaging.

A differential diagnosis of endogenous hyperinsulinemic hypoglycemia includes nesidioblastosis, also known as “non-insulinoma pancreatogenous hypoglycemia syndrome” in a clinical setting (Thompson et al., 2000). Histopathologically, this entity is defined as a diffuse hyperplasia of β-cells occurring usually in children (Yakovac et al., 1971). Recent evidence suggests that this pathology can also be demonstrated in adults, in particular after bypass surgery for morbid obesity (Service et al., 2005). In the previously mentioned series of patients (Christ et al., 2012), islet cell hyperplasia was diagnosed in three patients, two were positive on GLP-1R imaging and one was negative. Based on these preliminary data GLP-1R imaging does not appear to be an appropriate tool to diagnose or exclude islet cell hyperplasia. These findings are further supported by the recent evidence that the *in vitro* density of GLP-1R in pancreatic tissues of patients with nesidioblastosis after bypass surgery for morbid obesity is much lower than in benign insulinomas (Reubi et al., 2010). Recently, ^18^F-DOPA-PET has successfully been used to detect nesidioblastosis and benign insulinoma (Kauhanen et al., 2007). Although ^18^F-DOPA-PET may be helpful to diagnose nesidioblastosis, in benign insulinomas the tumor-to-background ratios are higher for ^111^In-DOTA-exendin-4 SPECT than for ^18^F-DOPA-PET (3.3 vs. 1.4), suggesting an increased sensitivity of targeting GLP-1Rs (Kauhanen et al., 2007; Christ et al., 2009) in benign insulinomas.

## SUMMARY AND CONCLUSION

Because of the massive GLP-1R overexpression in selected gastrointestinal tumors, GLP-1 and GLP-1R play an increasing role in endocrine gastrointestinal tumor management. Targeting GLP-1R with ^111^In-DOTA-exendin-4 or ^111^In-DPTA-exendin-4 offers a new approach that permits the successful localization of small benign insulinomas pre- and intraoperatively. Since virtually all benign insulinomas express GLP-1Rs and the preliminary clinical data are very encouraging, it is likely that this approach will affect the algorithm of pre- and intraoperative localization of suspected insulinoma.

In contrast to benign insulinomas, where the exact localization of the tumor is the main goal, the clinical challenge in malignant, metastasizing insulinomas is to define the extension of the disease and – if possible – offer a targeted therapy (peptide receptor radionuclide therapy, PRRT). Contrary to benign insulinomas, malignant insulinomas more often express sst2 receptors than GLP-1R. Importantly, one of the two receptors seems to be always expressed.

With regard to the increasing and successful use of GLP-1 analogs for diabetes therapy, it is worth keeping in mind that selected cancers can overexpress GLP-1R. While the functional role of these receptors in these tumors is not known yet, it may be safe to monitor patients with such tumors carefully during their GLP-1-analog-based diabetes therapy.

## Conflict of Interest Statement

The authors declare that the research was conducted in the absence of any commercial or financial relationships that could be construed as a potential conflict of interest.
